# Comparison of brief interventions in primary care on smoking and excessive alcohol consumption: a population survey in England

**DOI:** 10.3399/bjgp16X683149

**Published:** 2015-12-31

**Authors:** Jamie Brown, Robert West, Colin Angus, Emma Beard, Alan Brennan, Colin Drummond, Matthew Hickman, John Holmes, Eileen Kaner, Susan Michie

**Affiliations:** Health Behaviour Research Centre;; Health Behaviour Research Centre;; School of Health and Related Research, University of Sheffield, Sheffield.; Department of Clinical, Educational and Health Psychology, University College London, London.; School of Health and Related Research, University of Sheffield, Sheffield.; Institute of Psychiatry, Psychology and Neuroscience, King’s College London, London.; School of Social and Community Medicine, University of Bristol, Bristol.; School of Health and Related Research, University of Sheffield, Sheffield.; Institute of Health and Society, Newcastle University, Newcastle upon Tyne.; Department of Clinical, Educational and Health Psychology, University College London, London.

**Keywords:** alcohol drinking, brief advice, brief intervention, counselling, smoking

## Abstract

**Background:**

Brief interventions have a modest but meaningful effect on promoting smoking cessation and reducing excessive alcohol consumption. Guidelines recommend offering such advice opportunistically and regularly but incentives vary between the two behaviours.

**Aim:**

To use representative data from the perspective of patients to compare the prevalence and characteristics of people who smoke or drink excessively and who receive a brief intervention.

**Design and setting:**

Data was from a representative sample of 15 252 adults from household surveys in England.

**Method:**

Recall of brief interventions on smoking and alcohol use, sociodemographic information, and smoking and alcohol consumption patterns were assessed among smokers and those who drink excessively (AUDIT score of ≥8), who visited their GP surgery in the previous year.

**Results:**

Of 1775 smokers, 50.4% recalled receiving brief advice on smoking in the previous year. Smokers receiving advice compared with those who did not were more likely to be older (odds ratio [OR] 17-year increments 1.19, 95% confidence interval [CI] =1.06 to 1.34), female (OR 1.35, 95% CI =1.10 to 1.65), have a disability (OR 1.44, 95% CI = 1.11 to 1.88), have made more quit attempts in the previous year (compared with no attempts: one attempt, OR 1.65, 95% CI = 1.32 to 2.08; ≥2 attempts, OR 2.02, 95% CI =1.49 to 2.74), and have greater nicotine dependence (OR 1.17, 95% CI =1.05 to 1.31) but were less likely to have no post-16 qualifications (OR 0.81, 95% CI = 0.66 to 1.00). Of 1110 people drinking excessively, 6.5% recalled receiving advice in their GP surgery on their alcohol consumption in the previous year. Those receiving advice compared with those who did not had higher AUDIT scores (OR 1.17, 95% CI =1.12 to 1.23) and were less likely to be female (OR 0.44, 95% CI = 0.23 to 0.87).

**Conclusion:**

Whereas approximately half of smokers in England visiting their GP in the past year report having received advice on cessation, <10% of those who drink excessively report having received advice on their alcohol consumption.

## INTRODUCTION

Reducing the burden of tobacco smoking and excessive alcohol use is a public health priority^1^–^3^ that could be addressed by increasing the rate at which health professionals intervene opportunistically. Brief intervention to help patients stop smoking is an effective and cost-effective intervention.^4^–^6^ In the UK, the traditional model involves asking patients about their smoking, advising them to stop, and offering assistance in the form of a prescription for a pharmacological cessation aid or a referral to the NHS Stop Smoking Service.^7^ Guidelines from the National Institute for Health and Care Excellence (NICE) recommend that GPs assess the smoking status of patients at least once a year, advise smokers to stop, and record smoking status and any advice given.^5^

Brief intervention in primary care to help patients reduce excessive alcohol consumption also appears to be an effective and cost-effective measure.^8^–^10^ Such intervention requires screening individuals initially, and then providing those whose consumption is identified as high risk with structured brief advice or an extended brief intervention, and referring to specialist treatment services those identified to be at risk of dependent drinking.^8^,^11^ Implementation of screening and brief intervention in primary care across England has been advocated by NICE in recent UK guidance.^12^

Analyses of primary care databases indicate that the delivery of brief interventions for smoking may be relatively common: approximately 50% of smokers received advice in 2009.^13^,^14^ In contrast, previous assessments have suggested that clinicians rarely undertake screening and brief intervention to reduce excessive drinking.^15^–^17^ For example, an analysis of the General Practice Research Database indicated that GPs in England identified only an estimated 2% of patients who consumed alcohol excessively in 2003.^18^

There are several possible reasons for the difference between alcohol and smoking^19^–^25^ but one may be differences in financial incentives: there are substantial incentives to intervene on smoking in GP surgeries but less substantial opt-in arrangements for alcohol consumption.^26^ Specifically, GPs receive payments for recording the delivery of cessation advice through the primary incentivisation system for GPs in England, the Quality and Outcomes Framework (QOF).^14^,^27^,^28^ By contrast, QOF indicators do not currently cover unselected screening and brief intervention for excessive alcohol use,^29^ despite evidence that financial incentives can be effective in improving the delivery of screening and brief intervention.^17^,^30^–^32^ There is concern that this represents an important missed opportunity to reduce alcohol-related health harms at a population level.^33^ Beyond the QOF, there is a small financial incentive for using a validated tool to screen newly-registered patients.^34^,^35^ However, it is not part of the mandatory contract for performance management and, instead, practices have to opt in to offering an alcohol Directed Enhanced Service (DES) to claim the payment. Combined with the low level of remuneration and poor monitoring of outcomes, the result appears to be that the DES has had little effect on clinical behaviour.^17^,^36^ For example, among 75% of newly registered patients between 2007 and 2009 who had entries for alcohol consumption on a primary care database, only 9% were recorded as completing a validated screening questionnaire despite this financial incentive^29^ and NICE guidance to screen newly registered patients. Locally Enhanced Services can offer moderately increased financial incentives and this may explain some of the regional variation in screening that has been identified.^29^

**How this fits in**

Guidelines recommend intervening on both smoking and alcohol use opportunistically on a regular basis. In practice, analyses of GP recording databases indicate that a brief intervention on smoking is much more common than on alcohol. However, recent evidence suggests that the QOF incentives to promote intervention on smoking may have made the estimates for delivery based on recording databases somewhat unreliable. Therefore, this study used up-to-date and representative data from the perspective of patients to assess the prevalence of people who smoke or drink excessively, and who receive a brief intervention. This study confirmed that intervention on smoking appeared much more common than on alcohol: whereas approximately half of smokers in England visiting their GP in the previous year reported having received advice on smoking cessation, <10% of those who drink excessively reported having received advice on their alcohol consumption. In view of the substantial QOF incentives for the delivery of smoking brief interventions and the less substantial opt-in arrangements for alcohol brief interventions, this study adds to the evidence suggesting that more substantial incentives would be associated with greater delivery of a brief intervention.

Thus, there appears to be a large difference based on primary care databases between the delivery of brief intervention for smoking and alcohol,^13^,^14^,^18^ which may relate to the substantial financial incentives for smoking brief intervention but less substantial opt-in arrangements for alcohol.^26^ However, it is possible that figures derived from GP recording overestimate the delivery of smoking brief interventions because it is the recording rather than the ‘doing’ that is incentivised. Prior to the introduction of QOF incentives, there was a good correspondence between the rate of recording of GP advice and the proportion of patients recalling advice in the national Primary Care Trust Patient Surveys; since their introduction in 2004, however, the rate of recording has exceeded that of patient recall.^13^ The most recent estimate from the Primary Care Trust Patient Surveys is that 40% of smokers received cessation advice; however, this is of limited use as it represents a self-selected sample of patients who chose to return the survey. Estimates regarding the delivery of alcohol brief intervention are also likely to be inaccurate because they are often based on the rate at which GPs record screening, rather than conduct a brief intervention.

The aim of this study was to use up-to-date and representative data from the perspective of patients to assess the prevalence and characteristics of people who smoke or drink excessively, and who receive a brief intervention.

## METHOD

### Study design

Data were collected using cross-sectional household surveys of representative samples of the population of adults in England, conducted monthly between March 2014 and November 2014. The surveys are part of the ongoing Smoking Toolkit Study and Alcohol Toolkit Study, which are designed to provide tracking information about smoking, alcohol consumption, and related behaviours in England.^37^,^38^ Each month a new sample of approximately 1800 adults aged ≥16 years complete a face-to-face computer-assisted survey.

The sampling is a hybrid between random probability and simple quota. The first stage is to split England into 171 356 areas (each comprising approximately 300 households), stratified according to a geodemographic analysis of the population. Areas are then randomly allocated to interviewers, who conduct interviews within that area until the quota based on the probability of being at home is fulfilled. The method is superior to conventional quota sampling — where interviewers can select non-randomly from the whole population to meet quotas — because the output areas are randomly allocated and the scope for bias is limited to the choice of properties to approach non-randomly within the small output areas.

A response rate cannot be calculated because there is no definite gross sample, with units fulfilling the criteria of the quota being interchangeable. The sampling method has been shown to result in a sample that is nationally representative in its sociodemographic composition.^37^

### Study population

The study used aggregated data from responders, in the period from March 2014 to November 2014, who reported visiting their GP surgery in the previous year and either:

- smoked cigarettes or any other tobacco product daily or occasionally at the time of the survey or during the preceding 6 months; or
- drank alcohol excessively in the previous 6 months, indicated by a score of ≥8 on the Alcohol Use Disorders Identification Test (AUDIT).^39^

### Measures

Recall of smoking brief intervention among smokers and recall of alcohol brief intervention among people drinking excessively was assessed for the previous 12 months (a list of response items is available from the authors on request). Each group was separately classified into those who reported receiving at least a brief intervention (that is, including those who received a brief intervention followed by more intensive support), and those who did not. Responders were also asked questions that determined their:

- age;
- sex;
- occupation-based classification of socioeconomic status (‘social grade’):
  ▪ ABC1: higher and intermediate professional/managerial and supervisory, clerical, junior managerial/administrative/professional; or
  ▪ C2DE: skilled, semi-skilled, unskilled manual, and lowest-grade workers or unemployed);
- region in England:
  ▪ North: the north east, north west, and Yorkshire and the Humber;
  ▪ Central: East Midlands, West Midlands, and east of England; or
  ▪ South: London, south east, and south west);
- receipt of a post-16 educational qualification;
- children in the household;
- ethnicity; and
- disability.

Among smokers, past-year quit attempts and nicotine dependence (strength and time with urges to smoke^40^) were also assessed.

### Analysis

Weighted data were only used to estimate the delivery of alcohol and smoking brief interventions. Data were weighted using the rim (marginal) weighting technique to match an English population profile on the dimensions of age, social grade, region, tenure, ethnicity, and working status within sex. The dimensions were derived from the English 2011 census, Office for National Statistics 2013 mid-year estimates,^41^,^42^ and a random probability survey conducted in 2014 for the National Readership Survey (general details of the methodology of this survey are available elsewhere).^43^

The rim weighting was conducted in SPSS Quantum (version 5.8) and involved an iterative sequence of adjustments whereby a weight was applied to each responder such that the sample matched specified targets on a first dimension. In the next step, the data were then readjusted by an algorithm that sought to match the sample to a second dimension, while minimising distortion; this continued until the final dimension had been matched. This process was iterated until there was a good fit across the dimensions (indicated by the sum of root mean square differences between the sample and the specification across the dimensions being <0.005 multiplied by the overall unweighted sample size). More details on this method are available elsewhere.^44^,^45^

To examine the associations with patient characteristics, a series of univariable logistic regression models were constructed in which the receipt of a smoking brief intervention was regressed separately onto each patient characteristic. To examine the independent association after mutual adjustment, a multivariable logistic regression model was constructed including all patient characteristics and the month of survey. The patient characteristics entered in the univariable and multivariable models included the following dichotimised variables:

- sex;
- social grade;
- post-16 qualifications;
- children in the household;
- white ethnicity;
- disability; and
- excessive drinking (indicated by AUDIT score of ≥8).

Region was entered as a categorical variable with the North as the reference. The continuous variable age in years was transformed to reflect increases in the standard deviation of the sample (17 years) and was included with three more continuous variables:

- number of past-year quit attempts;
- time with urges to smoke; and
- strength of urges to smoke.

The linearity of the relationship between each continuous independent variable and the logit transformation of the dependent variable was indicated by the Box–Tidwell approach.^46^ This involved testing whether there was a significant interaction between the variable and its log transformation. There was a non-linear relationship with the number of past-year quit attempts, which was transformed into a categorical variable with 0 as the reference compared with one attempt, or ≥2 attempts.

Similar analyses were repeated to examine the univariable and multivariable associations between patient characteristics and the receipt of an alcohol brief intervention. Slightly different characteristics were examined to reflect that drinkers rather than smokers were under analysis. The characteristics assessed were the same except that excessive drinking, past-year quit attempts, time with urges to smoke, and strength of urges to smoke were excluded — instead, the continuous variable AUDIT score was included (since only those scoring ≥8 were used in this analysis), and smoking status was included as a categorical variable with ‘never smoker’ as the reference compared with ‘ex-smoker’ or ‘current smoker’. There was no evidence of a non-linear relationship with either of the continuous variables included in this analysis.

## RESULTS

An unweighted total of 15 252 adults aged ≥16 years were surveyed (the sociodemographic characteristics of the full sample are available from the authors); of these, 3043 (20.0%) were smokers and 1894 (12.4%) were drinking excessively. A total of 1889 (62.1%) smokers and 1116 (58.9%) people who drank excessively also reported visiting their GP; 1775 (94.0%) and 1110 (99.5%) respectively also had complete data on all relevant variables.

Of the unweighted sample of 1775, 925 (52.1%) smokers who visited their GP recalled having received a brief intervention for smoking. The weighted estimate was 50.4% (95% confidence interval [CI] = 48.0 to 52.8). Table 1 presents the associations between smoking, drinking, and sociodemographic characteristics and the receipt of a smoking brief intervention. In unadjusted univariable analyses, compared with those who received no intervention, those who did receive one were more likely to be older (odds ratio [OR] 1.19, 95% CI = 1.08 to 1.31), female (OR 1.28, 95% CI = 1.06 to 1.54), have a disability (OR 1.61, 95% CI = 1.26 to 2.05), to have made more quit attempts in the previous year (compared with no attempts: one attempt, OR 1.61, 95% CI = 1.29 to 2.01; ≥2 attempts, OR 2.07, 95% CI = 1.54 to 2.78), and have greater nicotine dependence (time with urges to smoke, OR 1.23, 95% CI = 1.14 to 1.33; strength of urges to smoke, OR 1.25, 95% CI = 1.15 to 1.36).

**Table 1.** Factors associated with receipt of brief intervention for smoking among smokers visiting their GP in the previous year

**Factor**	**Intervention, *n* = 925^a^**	**No intervention, *n* = 850**	**Unadjusted**			**Adjusted**		

			**Odds ratio**	**95% CI**	***P*-value**	**Odds ratio**	**95% CI**	***P*-value**
Mean age, years (SD)^b^	46.9 (16.8)	43.6 (17.2)	1.19	1.08 to 1.31	<0.01	1.19	1.06 to 1.34	<0.01
Female, *n* (%)	498 (53.8)	406 (47.8)	1.28	1.06 to 1.54	<0.05	1.35	1.10 to 1.65	<0.01
Lower social grade,^c^ *n* (%)	568 (61.4)	543 (63.9)	0.90	0.74 to 1.09	0.28	0.85	0.69 to 1.05	0.13

**Region, *n* (%)**								
North (ref)	360 (38.9)	334 (39.3)	–	–	–	–	–	–
Central	261 (28.2)	222 (26.1)	1.09	0.86 to 1.38	0.46	1.11	0.87 to 1.42	0.40
South	304 (32.9)	294 (34.6)	0.96	0.77 to 1.19	0.71	0.94	0.75 to 1.18	0.59
No post-16 qualification, *n* (%)	425 (45.9)	411 (48.4)	0.91	0.75 to 1.09	0.31	0.81	0.66 to 1.00	<0.05
Children in household, *n* (%)	306 (33.1)	312 (36.7)	0.85	0.70 to 1.04	0.11	0.96	0.76 to 1.21	0.74
White, *n* (%)	845 (91.4)	765 (90.0)	1.17	0.85 to 1.62	0.33	0.93	0.66 to 1.31	0.67
Disability, *n* (%)	205 (22.2)	128 (15.1)	1.61	1.26 to 2.05	<0.001	1.44	1.11 to 1.88	<0.01
Drinking excessively — AUDIT ≥8, *n* (%)	206 (22.3)	208 (24.5)	0.88	0.71 to 1.10	0.27	1.01	0.79 to 1.29	0.92

**Past-year quit attempts, *n* (%)**								
0 (ref)	505 (54.6)	576 (67.8)	–	–	–	–	–	–
1	271 (29.3)	192 (22.6)	1.61	1.29 to 2.01	<0.001	1.65	1.32 to 2.08	<0.001
≥2	149 (16.1)	82 (9.6)	2.07	1.54 to 2.78	<0.001	2.02	1.49 to 2.74	<0.001
Time with urge to smoke 0–5, mean (SD)	2.2 (1.3)	1.9 (1.2)	1.23	1.14 to 1.33	<0.001	1.17	1.05 to 1.31	<0.01
Strength of urge to smoke 0–5, mean (SD)	2.2 (1.1)	1.9 (1.1)	1.25	1.15 to 1.36	<0.001	1.08	0.96 to 1.23	0.21

a The 925/1775 does not precisely correspond with the 50.4% estimate presented in the main body as those data are weighted. The adjusted model includes all variables in the table and month of survey.

b Increase is per SD of the sample = 17 years of age.

c C2DE. AUDIT = Alcohol Use Disorders Identification Test. SD = standard deviation.

After mutual adjustment in a multivariable analysis, those receiving an intervention were more likely, than those who did not receive one, to be older (OR 1.19, 95% CI = 1.06 to 1.34), female (OR 1.35, 95% CI = 1.10 to 1.65), have a disability (OR 1.44, 95% CI = 1.11 to 1.88), to have made more quit attempts in the previous year (compared with no attempts: one attempt, OR 1.65, 95% CI = 1.32 to 2.08; ≥2 attempts, OR 2.02, 95% CI = 1.49 to 2.74), have greater nicotine dependence (time with urges to smoke OR 1.17, 95% CI = 1.05 to 1.31), and were less likely to have no post- 16 qualifications (OR 0.81, 95% CI = 0.66 to 1.00).

Of the unweighted sample of 1110, 76 (6.8%) people who drank excessively and visited their GP recalled having received an alcohol brief intervention; the weighted estimate was 6.5% (95% CI = 5.1 to 7.9). Table 2 presents the associations between drinking, smoking, sociodemographic characteristics, and the receipt of an alcohol brief intervention. In unadjusted univariable analyses, those who received an intervention, compared with those who did not, were more likely to be older (OR 1.35, 95% CI = 1.07 to 1.71), have ‘current’ compared with ‘never’ smoking status (OR 1.85, 95% CI = 1.05 to 3.26), and a higher AUDIT score (OR 1.17, 95% CI = 1.12 to 1.22); they were less likely to be female (OR 0.35, 95% CI = 0.19 to 0.65) and have children in the household (OR 0.50, 95% CI = 0.26 to 0.96). After mutual adjustment in a multivariable analysis, those receiving an intervention, compared with those who did not, had higher AUDIT scores (OR 1.17, 95% CI = 1.12 to 1.23) and were less likely to be female (OR 0.44, 95% CI = 0.23 to 0.87).

**Table 2.** Factors associated with receipt of brief intervention for alcohol among people drinking excessively visiting their GP in the previous year

**Factor**	**Intervention, *n* = 76^a^**	**No intervention, *n* = 1034**	**Unadjusted**			**Adjusted**		

			**Odds ratio**	**95% CI**	***P*-value**	**Odds ratio**	**95% CI**	***P*-value**
Mean age, years (SD)^b^	47.6 (16.2)	42.5 (17.9)	1.35	1.07 to 1.71	<0.05	1.32	0.99 to 1.78	0.06
Female, *n* (%)	12 (15.8)	362 (35.0)	0.35	0.19 to 0.65	<0.01	0.44	0.23 to 0.87	<0.05
Lower social grade,^c^ *n* (%)	35 (46.1)	450 (43.5)	1.11	0.69 to 1.77	0.67	1.08	0.63 to 1.86	0.79

**Region, *n* (%)**								
North (ref)	34 (44.7)	546 (52.8)	–	–	–	–	–	–
Central	15 (19.7)	191 (18.5)	1.26	0.67 to 2.37	0.47	1.18	0.59 to 2.36	0.64
South	27 (35.5)	297 (28.7)	1.46	0.86 to 2.47	0.16	1.30	0.73 to 2.32	0.37
No post-16 qualification, *n* (%)	25 (32.9)	319 (30.9)	1.10	0.67 to 1.80	0.71	0.72	0.40 to 1.29	0.27
Children in household, *n* (%)	11 (14.5)	261 (25.2)	0.50	0.26 to 0.96	<0.05	0.80	0.39 to 1.66	0.55
White, *n* (%)	72 (94.7)	1003 (97.0)	0.56	0.19 to 1.62	0.28	0.38	0.12 to 1.21	0.10
Disability, *n* (%)	14 (18.4)	114 (11.0)	1.82	0.99 to 3.36	0.05	1.19	0.59 to 2.43	0.63

**Smoking status, *n* (%)**								
Never smoke (ref)	21 (27.6)=	424 (41.0)	–	–	–	–	–	–
Ex-smoker	23 (30.3)	260 (25.1)	1.79	0.97 to 3.29	0.06	1.27	0.64 to 2.51	0.49
Current smoker	32 (42.1)	350 (33.8)	1.85	1.05 to 3.26	<0.05	1.14	0.59 to 2.20	0.69
AUDIT score 8–40, mean (SD)	15.5 (6.9)	11.1 (3.7)	1.17	1.12 to 1.22	<0.001	1.17	1.12 to 1.23	<0.001

a The 76/1110 does not precisely correspond with the 6.5% estimate presented in the main body as those data are weighted. The adjusted model includes all variables in the table and month of survey.

b Increase is per SD of the sample = 17 years of age.

c C2DE. AUDIT = Alcohol Use Disorders Identification Test. SD = standard deviation.

## DISCUSSION

### Summary

Smokers in England who reported visiting their GP appeared substantially more likely to receive advice about their smoking status than people drinking excessively were about their alcohol consumption: 50% of smokers recalled receiving a brief intervention on smoking, whereas <10% of those drinking excessively recalled having received a brief intervention on alcohol. Smokers receiving advice, compared with those who did not, were more likely to be older, female, have a disability, have made more quit attempts in the previous year, have greater nicotine dependence, but be less likely to have no post-16 qualifications. People drinking excessively and receiving advice had higher AUDIT scores and were more likely to be male than those who did not receive advice, but no other associations were clearly established.

### Strengths and limitations

The present findings are similar to estimates of delivery of brief interventions derived from primary care databases.^13^,^14^,^29^ A major strength of this study is that these figures are up to date and from the perspective of patients identified from a large representative sample of the English population. A consequent limitation is that the findings may be inaccurate because patients either forgot about receiving an intervention or misjudged the time period assessed. Although self-reported data are subject to such recall bias, there is evidence that financial incentives lead to improved recording — rather than improved delivery — of brief interventions, so it is important to use data from both GP records and patients when forming a judgement.^13^

Another strength of the study arises because the delivery of alcohol brief interventions is often not recorded by clinicians, but generally indirectly estimated from the rate at which patients are screened for excessive alcohol consumption.^18^,^29^ Future research could compare estimates more directly by surveying a representative sample of the population, and seeking permission and details to allow the patient records of responders to be identified.

This study is also limited by assessing only the association between receipt of brief interventions and patient characteristics; the receipt of brief intervention is also likely related to GP characteristics and those of their surgeries.^19^–^25^

### Comparison with existing literature

People with higher compared with lower AUDIT scores were more likely to recall a brief intervention than to not: the associated odds of recalling a brief intervention were 17% greater for every point increase above 8 on the AUDIT scale (OR 1.17). This finding is consistent with a previous analysis which indicated that GPs were more likely to identify dependent drinkers compared with those who were drinking at lower, but still harmful, levels.^18^ GPs have also been found to identify an alcohol use disorder less often in younger people and dependence less often in females,^18^ which is consistent with the associations between age, sex, and receipt of brief intervention in the study presented here.

The association for smokers with age, sex, previous quit attempts, and dependence reflects the profile of treatment-seeking smokers and is likely to be related to GPs focusing on smokers who express an interest in stopping.^47^ These findings suggest GPs are not yet following the latest national guidance from the National Centre for Smoking Cessation and Training (NCSCT) in England, which recommends that they go straight to the offer of support, rather than assess a patient’s interest in quitting.^6^,^48^

The association with disability may reflect the greater incentives for brief intervention among smokers with chronic health conditions.^28^ Previous research has found that smokers from more disadvantaged socioeconomic backgrounds want and try to quit as much as other smokers but find it more difficult.^49^ This difficulty suggests the marginal benefit from brief intervention recommending support would be greater for this group. However, the current study suggests that smokers without post-16 qualifications are less likely to receive an intervention, which is concerning in terms of health inequalities.

### Implications for practice

The UK government’s alcohol strategy was criticised by Alcohol Health Alliance UK for its failure to implement a QOF indicator for screening and brief intervention for excessive alcohol consumption.^33^ In view of the substantial QOF incentives for the delivery of smoking brief interventions and the less substantial opt-in arrangements for alcohol brief interventions, this study adds to the evidence suggesting that more substantial incentives are likely to be associated with greater delivery of brief intervention.^17^,^26^,^30^–^32^

There are other reasons why the delivery of smoking and alcohol brief interventions may differ; for example, excessive alcohol consumption takes longer to establish than smoking because it requires screening, motivation to change is weaker among those who drink excessively than among smokers in England,^50^,^51^ and drinking advice may be less straightforward because reduction is often the goal rather than abstinence. However, the magnitude of the difference suggests that a number of factors could be important and scope remains for enhanced financial incentives to have a significant impact.

The focus of this article is alcohol and tobacco control in England. Although it is true that the effectiveness of any policy will always depend on a variety of local, cultural, and contextual factors,^52^ studying what happens in England with contrasting financial incentives for the delivery of smoking and alcohol brief interventions could provide an indirect indication as to what is achievable in other countries.

There is wide debate about what constitutes an effective alcohol brief intervention.^11^,^53^–^55^ An enhanced incentive scheme for alcohol brief intervention in England — such as a QOF indicator for unselected screening and brief intervention delivery — may maximise effectiveness and cost-effectiveness by being structured to encourage brief screening followed by simple feedback and written information.^53^ The simple behaviour change technique of self-monitoring has been shown to be effective for reducing alcohol consumption and would be a relatively easy technique for GPs to use with their patients.^56^ Including this would meet the recent recommendation of the House of Lords Science and Technology Select Committee to include more explicit advice in guidance on how behaviour change techniques can be applied to reducing excessive alcohol consumption.^57^

Any improvement to incentive schemes would likely be synergistically enhanced by the simultaneous provision of additional training on how to deliver brief interventions.^32^ NICE guidelines recommend that all health and social care professionals should, as a minimum, be able to deliver a very brief intervention.^58^ The NCSCT offers an online brief advice module, which increases the frequency and quality of smoking brief interventions,^59^ while training on the formulation of specific action plans increases the rate of asking about smoking;^60^ future research could assess whether such advice modules and action plans have a similar effect on alcohol brief intervention.
